# How often do we need to update PEEP setting during prone positioning in ARDS?

**DOI:** 10.1186/s13054-024-04847-w

**Published:** 2024-02-26

**Authors:** Ling Sang, Zhimin Lin, Zhanqi Zhao

**Affiliations:** 1 State Key Laboratory of Respiratory Diseases, Guangzhou Institute of Respiratory Health, The First Affiliated Hospital of Guangzhou Medical University, Guangzhou, China; 2 Department of Critical Care Medicine, The First Affiliated Hospital of Guangzhou Medical University, Guangzhou, China; 3 Guangzhou National Laboratory, Guangzhou, China; 4 School of Biomedical Engineering, Guangzhou Medical University, Guangzhou, China; 5 Department of Critical Care Medicine, Peking Union Medical College Hospital, Chinese Academy of Medical Sciences, Beijing, China; 6 Institute of Technical Medicine, Furtwangen University, Villingen-Schwenningen, Germany

## To the Editor

Patients with acute respiratory distress syndrome (ARDS) require mechanical ventilation. Personalized lung protective ventilation strategy with low tidal volume, adequate positive end-expiratory pressure (PEEP) and limited plateau pressure helps to reduce ventilator-induced lung injury and improves ARDS survival [1]. Prone positioning improves dorsal ventilation and ventilation-perfusion matching in ARDS [2, 3]. Gravitational influence is similar in both supine and prone positions. PEEP should be optimized in prone position as well. Currently, the application of PEEP titration and the frequency vary from center to center. A previous study suggested that PEEP setting may need to be changed post-pronation to achieve better respiratory system compliance (Crs) [4]. No study so far investigates the change of PEEP setting in prone positioning for  > 24 h. We conducted a preliminary study to examine the trend of optimal PEEP and the resulting physiological parameters in the course of prone positioning up to 42 h. The investigated parameters included Crs, mechanical power and the ratio of partial pressure of oxygen in arterial blood (PaO_2_) to the fraction of inspiratory oxygen concentration (FiO_2_).

Consecutive ARDS patients presenting with PaO_2_/FiO_2_ < 150 mmHg while on invasive mechanical ventilation with PEEP > 5 cmH_2_O were screened for eligibility. Patients who underwent their initial prone positioning session for a duration of at least 30 h, as determined by the attending physician, were included in the study. PEEP titration was conducted at a discrete 6-h intervals (T_SB_, supine; T_P0_, after proning the patient; T_Px_, x = 6, 12, 18, …hours after prone position; T_SA6_, 6 h after turning the patients back to supine position). The decremental PEEP titration began at 16 cmH_2_O and progressively decreased to 6 cmH_2_O in 2 cmH_2_O increments every 2 min. Optimal PEEP was selected according to the compromise of regional overdistension and collapse assessed by electrical impedance tomography (EIT, Pulmovista 500, Dräger Medical, Lübeck, Germany) [5]. A silicon belt with 16 electrodes was placed around the patient’s thorax transversely at the fourth–fifth intercostal space according to the manufacturer’s instructions. The exact placement of the electrode belt was marked so that at each measurement time point, the EIT measurement planes were similar. Lung mechanics and blood gasses analysis were recorded.

**Fig. 1:**
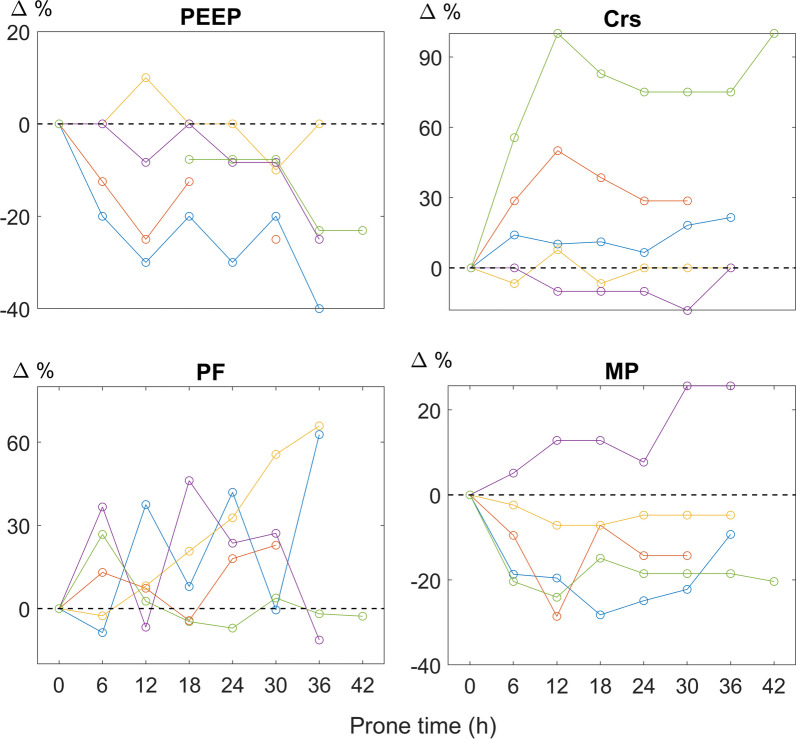
Summary of the trend of optimal PEEP, respiratory system compliance (Crs), PaO_2_/FiO_2_ (PF) and mechanical power (MP) during prone position. The values are normalized to time point 0 immediately measured after prone position started

A total of five patients were included in the analysis. The average PaO_2_/FiO_2_ was 111.6 mmHg (max 148, min 71) at the supine position before proning started. One patient was in prone position for 30 h, three patients for 36 h, and one patient for 42 h. Optimal PEEP, Crs, PaO_2_/FiO_2_ and mechanical power during prone position were normalized to those values at T_P0_, and the trends are summarized in Fig. 1. EIT-guided optimal PEEP decreased progressively in four patients (Fig. 1 top left). Crs increased in three patients, decreased in one and remained in one (Fig. 1 top right). Improvements in PaO_2_/FiO_2_ and mechanical power were found in most of the patients (Fig. 1 bottom). However, in one patient, mechanical power increased gradually with a drop in PaO_2_/FiO_2_ at T_P12_ and T_P36_. Nevertheless, compared to those at T_SB_, PaO_2_/FiO_2_ were higher at T_SA6_ in all studied patients (average increase 74 mmHg). Improvement was also found in mechanical power at T_SA6_ in all patients (average decrease 2.0 J). Crs was increased in three patients (average 9.1 ml/cmH_2_O) and remained unchanged in the other two patients. No adverse events were noted during prone position in the studied subjects.

The current study presents a summary of initial findings from five patients with moderate–severe ARDS during prolonged prone positioning. Our observations indicate a dynamic shift in lung mechanics and oxygenation, underscoring the crucial need for timely ventilator adjustments throughout extended prone periods. While the standard proning duration is typically recommended at around 16 h, the concept of extra-long prone positioning emerged during the COVID-19 pandemic when healthcare staff faced constraints. Contrary to conventional practice, we observed instances where patients exhibited ongoing improvements in lung function and oxygenation even after 30 h of prone position. The benefits persisted 6 h post-supination. This prompted our current study to document the physiological variations during prolonged proning. The optimal duration for prone positioning hinges significantly on individual responses to ventilation-perfusion adjustments and disease progression [3]. In our study, a substantial deterioration in mechanical power post-30-h proning was noted in one patient, with a concurrent drop in PaO_2_/FiO_2_ at T_P36_ (Fig. 1 bottom purple lines). Despite achieving similar ventilation homogeneity at lower PEEP level, extended prone positioning might not be advisable. The principal limitation of this study lies in its small sample size, precluding robust statistical analyses. Nonetheless, the personalized trends in optimal PEEP, lung mechanics and oxygenation were clearly illustrated. Future investigations should explore the link between personalized proning durations, ventilator adjustments and patient outcomes such as ventilator-free days and mortality rates, aiming to provide valuable insights for clinical practice.

## Data Availability

The datasets used and/or analyzed during the current study are available from the corresponding author on reasonable request.

## Abbreviations

ARDS: Acute respiratory distress syndrome
Crs: Respiratory system compliance
EIT: Electrical impedance tomography
MP: Mechanical power
PaO_2_/FiO_2_: The ratio of partial pressure of oxygen in arterial blood to the fraction of inspiratory oxygen concentration
PEEP: Positive end-expiratory pressure
